# DCPS is a synthetic lethal therapeutic target in acute myeloid leukemia expressing low levels of FHIT

**DOI:** 10.1038/s41375-025-02661-z

**Published:** 2025-06-26

**Authors:** Francesca Grassi, Madhurendra Singh, Simon Moussaud, Gabriela Vazquez Rodriguez, Zaheer Ali, Kirsten Janssen, Mathilde Cheray, Elory Leonard, Martin Andersson, Angelo de Milito, Hong Qian, Julian Walfridsson, Andreas Höglund

**Affiliations:** 1 Sprint Bioscience AB, Huddinge, Sweden; 2 Department of Medicine Huddinge, Centre for Haematology and Regenerative Medicine, Karolinska Institute, Stockholm, Sweden; 3 BioReperia AB, Linköping, Sweden; 4 NeoTargets AB, Huddinge, Sweden

**Keywords:** Acute myeloid leukaemia, Differentiation

## To the Editor:

**Fig. 1:**
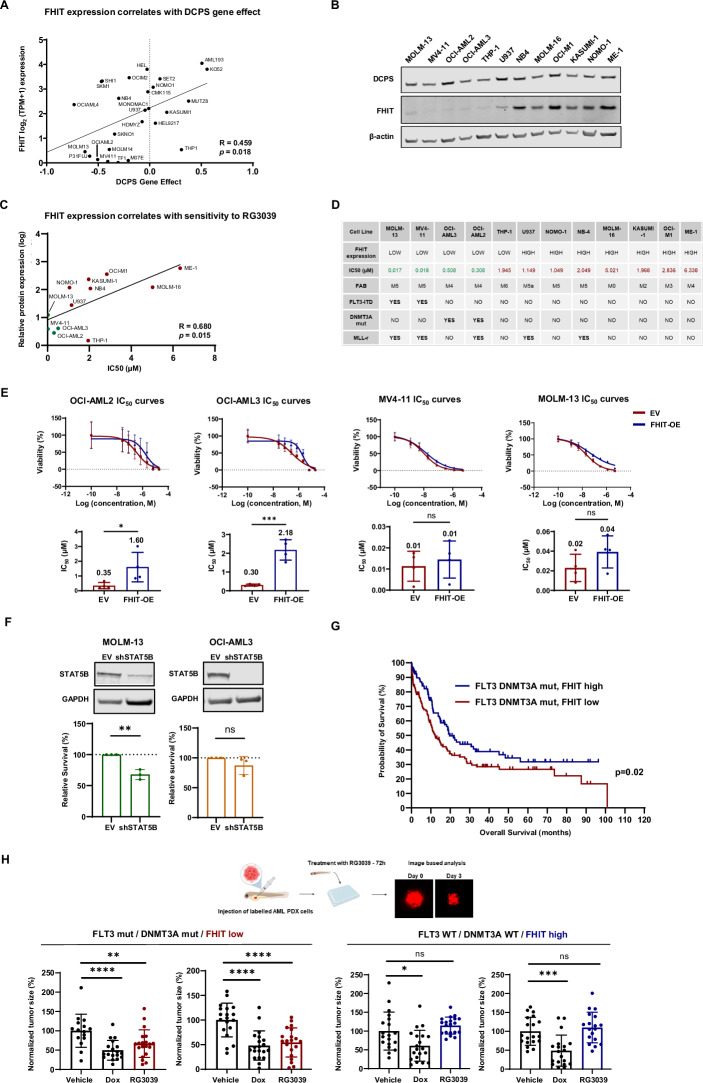
FHIT expression along with mutations in DNMT3A and FLT3 modulates the response of AML to DCPS inhibition. **A** Scatterplot showing the Pearson correlations between DCPS dependency score (Chronos Gene Effect) and FHIT expression (log_2_(TPM + 1)) in AML cells from DepMap (24Q4); *p = 0.018, *R* = 0.459. **B** Immunoblot analyses of DCPS and FHIT expression in AML cell panel. β-actin was used for signal normalization (*n* = 2). **C** Scatter plot showing Pearson correlations between IC50 values of AML cell lines treated with RG3039 and relative FHIT protein expression (normalized to β-actin, log-transformed). In green cells with low FHIT expression and sensitive to DCPS inhibition, in red more resistant cells with higher FHIT expression; *p = 0.015, *R* = 0.680 (*n* = 3). **D** Schematic summary of genetic and clinical classification of AML cell panel. FHIT protein expression was quantified in Fig. 1B and Supplementary Fig. 1A. French-American-British (FAB) classification of AML is used. FLT3-ITD is a common AML mutation (~30% cases) and a prognostic indicator of disease outcome. DNMT3A is commonly mutated in AML (~20% cases). **E** Nonlinear regression curves of viability in AML cells over-expressing FHIT compared to EV. Bar chart shows mean ± SD of IC50 values (*n* = 4); *p < 0.05, ***p < 0.001, unpaired *t* test. **F** Immunoblot analysis of shRNA knock-down of STAT5B in MOLM-13 and OCI-AML3 cells. Bar chart shows mean ± SD of relative viability in STAT5B-KD AML cells compared to EV (*n* = 3). **G** Kaplan-Meier survival analysis of AML patients carrying FLT3 and DNMT3A mutations with low (red line) or high (blue line) FHIT expression (z-score relative to log_2_ RNA Seq RPKM). Log-rank (Mantel-Cox) test was used to determine significant differences, *p = 0.02. **H** Normalized tumor size of patient-derived AML xenograft models in Zebrafish (ZTX^®^) treated with doxorubicin and IC90 dose of RG3039 for 72 h, expressed as mean ± SD of percent change compared to vehicle (*n* = 20); *p < 0.05. **p < 0.01, ***p < 0.001, ****p < 0.001, unpaired *t* test.

For a therapy to be successful in the clinic, efficacy and safety are paramount. The identification of viable biomarkers is also advantageous for increasing treatment success. Recently, the pyrophosphatase DCPS (Decapping scavenger enzyme) was identified and validated as a potential therapeutic target in Acute Myeloid Leukemia (AML), demonstrating that pharmacological inhibition of DCPS is detrimental for AML cells but dispensable for normal haematopoiesis [1]. The main function of DCPS is to remove the potentially toxic accumulation of mRNA (M7GTP) caps present on the 5′ end of mRNA transcripts by hydrolysing the triphosphate linkage of the cap structure, yielding 7-methylguanosine monophosphate and nucleoside diphosphate [2]. FHIT (Fragile Histidine Triad Diadenosine Triphosphatase) has overlapping substrate specificity with DCPS and is also able to degrade M7GTP cap structures in the 3´-5´mRNA decay pathway [3]. We sought to investigate the possibility of stratifying DCPS targeted therapy by selecting patients based on expression of FHIT, testing the hypothesis that FHIT would negate the therapeutic window of a DCPS small molecular inhibitor. We first investigated the potential connection by using data in the Dependency Map (DepMap) (24Q4 Public), where we found that AML cell lines expressing low levels of FHIT were more sensitive to DCPS knock-out (Fig. 1A). RG3039 is a potent and selective small molecule inhibitor against DCPS with proven clinical safety [4]. To validate the interdependency data between DCPS and FHIT in DepMap we used RG3039 to pharmacologically inhibit DCPS in a panel of AML cell lines. We first measured the expression of FHIT and DCPS using WB and qRT PCR (Fig. 1B and Supplementary Fig. 1A, B). All cell lines had uniformly high expression of DCPS but varied in the expression of FHIT. By testing this panel for sensitivity against RG3039 and correlating the IC50 from each cell line to FHIT expression, we identified four cell lines that clustered together showing both sensitivity towards DCPS inhibition and low expression of FHIT (MV4-11, MOLM-13, OCI-AML2 and OCI-AML3) (Fig. 1C and Supplementary Fig. 1C, D). Next, we used CETSA® to show that RG3039 does not directly bind to FHIT in cells but does show strong target engagement with DCPS (Supplementary Fig. 1E), thus concluding that FHIT levels modulate the response to pharmacological inhibition of DCPS. To further prove that FHIT levels reversely correlate with the response to pharmacological inhibition of DCPS, we knocked down FHIT using shRNA in RG3039 resistant OCI-M1 cells and as expected based on the above data the cells became more sensitive to DCPS inhibition (Supplementary Fig. 1F). Further subclassification of the AML cell panel identified additional biomarkers besides FHIT that could potentially modulate the response to DCPS inhibition, including mutations in DNMT3A and FLT3 internal tandem duplications (ITD) [5] (Fig. 1D). To definitively prove that FHIT can reduce the response to DCPS inhibition, we introduced FHIT via lentiviral transduction in RG3039 sensitive cell lines MV4-11 (FLT3-ITD), MOLM-13 (FLT3-ITD), OCI-AML2 (DNMT3A mut) and OCI-AML3 (DNMT3A mut) (Supplementary Fig. 1G). When testing these cell lines for sensitivity to DCPS inhibition using RG3039 only DNMT3A mutant OCI-AML2 and OCI-AML3 cells overexpressing FHIT showed reduced sensitivity with several fold increase in IC50 value compared to cell lines transduced with an empty vector (EV) (Fig. 1E). STAT5 is a direct target of FLT3 and FLT3 mutated AML are dependent on STAT5 for survival [6]. STAT5B was downregulated by DCPS inhibition in a dose dependent manner irrespective of FHIT expression in MOLM-13 cells (Supplementary Fig. 1H) and knockdown of STAT5B in MOLM-13 and OCI-AML3 cells only compromised the viability of MOLM-13 cells (Fig. 1F). Since FHIT degrades cap structures similarly to DCPS, these data indicate a M7GTP cap independent mechanism of cell death in FLT3 mutated AML following DCPS inhibition. M7GTP cap scavenging independent functions of DCPS including turnover of miRNAs have been shown [7], providing a possible explanation behind the bias towards FHIT mediated rescue of DNMT3A mutant AML cells upon DCPS inhibition.

DNMT3A and FLT3 mutations are some of the most common driver mutations in AML and frequently co-occur [8]. In patients carrying AML mutated for DNMT3A and FLT3 (*2022* OHSU repository in cBioPortal), low expression of *FHIT* correlates with reduced overall survival, suggesting that these patients could potentially benefit from DCPS targeted therapy (Fig. 1G). To challenge this hypothesis, we injected 2-day old Zebrafish embryos with 2 primary AML PDX samples with FLT3/DNMT3A mutations (*FHIT* low expression) and 2 primary AML PDX samples with WT FLT3/DNMT3A (*FHIT* high expression) to serve as controls (Supplementary Fig. 1I). The IC90 dose of RG3039 (MOLM-13/MV4-11—Supplementary Fig. 1C) was added directly to the water of the Zebrafish and the relative tumor size was measured after 3 days of DCPS inhibition. Only the FLT3/DNMT3A mutant low *FHIT* expressing AML PDX cells showed significant tumor reduction, whereas the control AML PDX samples were completely resistant to treatment (Fig. 1H). Despite physiological differences from mammals, the zebrafish model serves as a valuable predictive system for therapeutic response in cancer [9], with our data demonstrating its potential to guide patient stratification for DCPS inhibition therapy in AML based on DNMT3A, FLT3, and FHIT status.

**Fig. 2:**
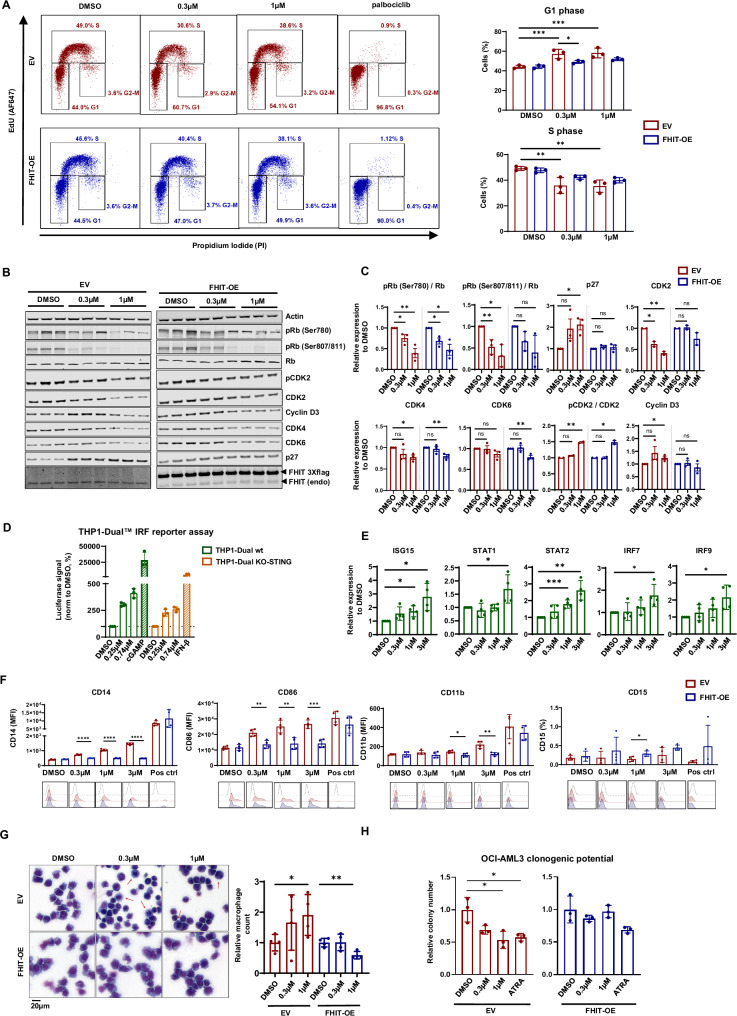
DCPS inhibition induces cell cycle arrest and release of differentiation block in AML with low levels of FHIT. **A** Flow cytometry analysis of cell cycle upon 24-hour treatment with RG3039 using EdU-Alexa Fluor 647 and PI incorporation. Palbociclib was used as positive control to block S phase. Percentage of EV (red) and FHIT-OE (blue) OCI-AML3 in G1 and S phase is shown on the right as mean ± SD (*n* = 3). **B** Immunoblot analyses of G1 R point regulators following treatment with RG3039 for 24 h. Relative expression to DMSO control is normalized to β-actin and shown in **C** as mean ± SD (*n* = 3). **D** Bar chart of luciferase signal induced in THP1-Dual™ wild-type (green) and KO-STING (orange) reporter cells upon treatment with RG3039 for 24 h. Data are shown as mean ± SD of signal normalized to DMSO (*n* = 3). **E** Bar chart shows mean ± SD of THP-1 gene expression for *ISG15*, *STAT1*, *STAT2*, *IRF7* and *IRF9* after 24-hour treatment with RG3039 relative to DMSO (*n* = 4). Expression was normalized to *RPL13A*, *HPRT1*, *ACTB*, *TBP* housekeeping genes; *p < 0.05, **p < 0.01, ***p < 0.001, unpaired *t* test. **F** Flow cytometry analyses of surface markers for myeloid differentiation in OCI-AML3 EV and FHIT-OE. Data are presented as mean ± SD of Mean Fluorescent Intensity (MFI) or percentage of positive cells (%) (*n* = 4). **G** Giemsa May-Grünwald stained representative images of OCI-AML3 treated with RG3039 for 5 days. Macrophages are indicated by red arrows and are only present in EV cell line upon treatment. Scale bar is 20 µm. Quantification is shown on the right as mean ± SD of macrophage ratio compared to DMSO (*n* = 3). **H** Relative number of colonies to DMSO control formed by EV or FHIT-OE OCI-AML3 treated with RG3039 for 5 days. Data are shown as mean ± SD (*n* = 3). For all panels significance was calculated using unpaired *t* test, *p < 0.05; **p < 0.01; ***p < 0.001; ****p < 0.0001.

To understand the phenotypic differences introduced by FHIT expression we treated OCI-AML3 EV and FHIT-OE cells with RG3039 and pulsed them with EdU. EV cells responded with an apparent cell cycle exit, revealing a robust increase of cells in G1 and much fewer cells in S-phase. The cells overexpressing FHIT displayed a much milder response, with small changes in G1 and S phase distribution (Fig. 2A). Immunoblotting for regulators of G1 phase cell cycle transition revealed reduction of Rb phosphorylation in both OCI-AML3 EV and FHIT-OE OCI-AML3 cells while RG3039 treatment induced an increase in the CDK inhibitor p27 and reduction in CDK2 only in the EV cell line, explaining the robust cell cycle exit observed (Figs. 2B, C). We also observed an EV specific reduction in E2F target genes *CDC6* and *MCM3* [10] (Supplementary Fig. 2A). Gene expression data (*2022* OHSU repository in cBioPortal) also indicated a strong correlation between *DCPS* and both *MCM3* and *CDC6*, further strengthening the connection between DCPS and regulation of the G1/S transition (Supplementary Fig. 2B). The toxic accumulation of the M7GTP cap upon DCPS inhibition could potentially mislocalize and reduce the active pool of components of the cap binding complex (CBC) [11]. The CBC complex is vital for pre-mRNA splicing [12]. We observed a dose dependent reduction of CBC member CBP80 in the nuclear compartment of OCI-AML3 EV cells, but not in cells overexpressing FHIT (Supplementary Fig. 2C, D). Miss-splicing of RNA has previously been reported in AML upon DCPS inhibition [1], and such viral-mimic double stranded RNA stress could induce interferon mediated cell cycle arrest [13]. To test for RNA stress induction following DCPS inhibition, we treated THP1-Dual™ Reporter WT or STING KO cells with RG3039 and monitored the expression of IRF (Interferon Regulatory Factor) mediated signal induction. In both cell lines, inhibition of DCPS significantly increased the IRF pathway induction (Fig. 2D). Since STING is responsible for signal transduction downstream of cytosolic DNA accumulation, the data suggests that the IRF-mediated signal is primarily an effect of RNA accumulation. DCPS inhibition also led to a gene induction (*ISG15, STAT1, STAT2, IRF7* and *IRF9*) that is consistent with viral-mimic RNA stress [14] (Fig. 2E). G1 blockade following RNA stress could signal for cells to differentiate. Differentiation blockade is a major hallmark of AML pathology, and reversal of this phenotype would have considerable therapeutic benefits. Therefore, we treated OCI-AML3 cells with RG3039 and monitored the expression of differentiation markers CD11b, CD14, CD15 and CD86 using flow cytometry. CD14 and CD86 were upregulated in a dose dependent manner in EV cells but not when FHIT was overexpressed (Fig. 2F). Treatment with RG3039 also increased Annexin V staining, an early indicator of apoptosis, without reaching statistical significance. Strikingly, this seemed to be completely prevented in cells overexpressing FHIT (Supplementary Fig. 2E), suggesting that FHIT blocks differentiation and apoptosis upon DCPS inhibition in DNMT3A mutant AML. To investigate the fate of the differentiating cells, we treated OCI-AML3 EV and FHIT-OE cells with RG3039 and stained them with May Grünwald Giemsa. Upon treatment with RG3039 OCI-AML3 EV cells were enriched for macrophages (Fig. 2G) and showed reduced colony forming potential in MethoCult™ media (Fig. 2H and Supplementary Fig. 2F). Such phenotypes were abolished upon FHIT overexpression. These data show the potential of DCPS inhibition to overcome the differentiation blockade in AML, given that FHIT activity is concomitantly low.

DCPS is a drug target with an excellent safety profile supported by both preclinical and clinical data. *FHIT* promoter methylation is found in 14% of AML and 50% of MDS patients and increases upon relapse in AML [15]. We propose that FHIT expression, along with mutations in FLT3 and DNMT3A are viable biomarkers to stratify DCPS targeted therapy. Collectively, our data demonstrate that DCPS is a synthetic lethal therapeutic target in AML expressing low levels of FHIT.

## Supplementary information

Supplementary Information
