# Supplementary material for: DCPS is a synthetic lethal therapeutic target in acute myeloid leukemia expressing low levels of FHIT
Source: Leukemia. 2025 Jun 26;39(8):2021–5. doi: 10.1038/s41375-025-02661-z (PMC12310507; doi:10.1038/s41375-025-02661-z)

Supplementary Information

1. **Supplementary Materials and Methods**
2. **Supplementary Figure Legends**
3. **Supplementary Figures**

Methods

AML Cell Culture

NOMO-1 cell line was obtained by the American Type Culture Collection (ATCC) (Middlesex, UK); MV4-11, NB-4, MOLM-13, U937, THP-1, KASUMI-1, MOLM-16, ME-1, OCI-AML2, OCI-AML3 and OCI-M1 were obtained from DSMZ (Braunschweig, Germany). All cell lines were authenticated via STR profiling. NOMO-1, MV4-11, NB-4, MOLM-13, U937, THP-1 cell lines were grown in RPMI 1640 (Thermo Fisher Scientific, Waltham, MA, USA) supplemented with 10% FBS Heat Inactivated (HI) (Thermo Fisher Scientific); KASUMI-1, MOLM-16, ME-1 cell lines were grown in the same RPMI 1640 supplemented with 20% FBS HI. OCI-AML2 and OCI-AML were grown in MEM-α (Thermo Fisher Scientific) supplemented with 10% FBS HI. OCI-M1 was cultured in IMDM (Thermo Fisher Scientific) supplemented with 10% FBS HI. Cells were periodically tested for Mycoplasma contamination using MycoAlert® PLUS Mycoplasma Detection Kit (Lonza, Basel, Switzerland).

Cell Viability Assay

To calculate the cell viability, cells were plated in 96-well plates at 5000 or 1000 cells per well in 50µL medium, then mixed with the same volume of 2X concentrations of DCPS inhibitor RG3039 (MedChemExpress, Princeton, NJ, USA), serially diluted from 20 or 5µM in a 7-step dilution. DMSO and Benzethonium Chloride (BzCl) were used as negative and positive controls, respectively. CellTiter® Glo 2.0 Cell Viability Assay (Promega, Madison, WI, USA) was added after 72 hours following the manufacturer's protocol, and luminescence was measured with CLARIOstar PLUS (BMG Labtech, Ortenberg, Germany). Data was analysed using GraphPad Prism software (v.10.0.3). Survival curves were obtained by non-linear regression of log-transformed and normalized values using a variable slope model. IC_{50} values were automatically calculated by the software.

Immunoblot assay

After treatment or transfection, cells were lysed on ice for 30 minutes, vortexing every 10 minutes; then, ultracentrifugation at 20,000 rcf for 15 minutes at 4°C was performed. Supernatant was used for protein quantification and Western Blot. Pierce BCA protein Assay kit (Thermo Fisher Scientific) was used to quantify protein following manufacturer's protocol with the only exception that 10µL of diluted sample and standards were used. Absorbance was measured using CLARIOstar PLUS (BMG Labtech) and

data analysed in the MARS software (BMG Labtech). Blank-corrected values were quantified using the standard curve and relative concentrations in µg/µL. Variable protein amount was resuspended in 4X orange Protein Samples Loading Buffer (LI-COR Biosciences, Lincoln, NE, USA) supplemented with 40mM DTT (Thermo Fisher Scientific), denatured at 95°C for 5 minutes, then resolved in Bis-Tris 4-12% gels (Thermo Fisher Scientific) using 1X MES running buffer (Thermo Fisher Scientific). Nitrocellulose membranes (Thermo Fisher Scientific) were used for transfer using the iBlot™ 2 Dry Blotting System (Thermo Fischer Scientific). Membranes were blocked in Intercept™ (TBS) Blocking Buffer (LI-COR Biosciences) for 60 minutes room temperature (RT) and incubation with primary antibody was performed overnight (ON) at 4°C. After 3x washing steps with TBS-T supplemented with 0.05% Tween-20 (Sigma-Aldrich, Bulington, MA, USA), membranes were incubated with secondary antibodies for 60 minutes. Membranes were developed on the LI-COR Odysseus CLx (LI-COR Biosciences) using auto scan and images were analysed using Empiria Studio® Software (LI-COR Biosciences, v.2.2) or Image Studio (LI-COR Biosciences). Primary antibodies used are listed below:

DCPS	Santa-Cruz Biotechnologies	sc-393226
FHIT	Santa-Cruz Biotechnologies	sc-390481
Rb	Santa-Cruz Biotechnologies	sc-102
Phospho-Rb (ser780)	Cell Signaling Technology	9307T
phospho-RB (Ser807/811)	Cell Signaling Technology	8516S
CDK6	Santa-Cruz Biotechnologies	sc-7961
CDK4	Cell Signaling Technology	12790S
CDK2	Santa-Cruz Biotechnologies	sc-6248
Phospho-CDK2	Cell Signaling Technology	2561S
Cyclin D3	Cell Signaling Technology	2936T
$P27^{KIP1}$	Cell Signaling Technology	3686S
CBP80	Santa-Cruz Biotechnologies	sc-271304
STAT5B	Cell Signaling Technology	34662S
β-actin	Cell Signaling Technology	4970S
GAPDH	Cell Signaling Technology	2118S
PCNA	Cell Signaling Technology	13110S

Histone H3	Cell Signaling Technology	14269S
α-tubulin	Cell Signaling Technology	2144S

Secondary antibodies: IRDye® 800CW or 680RD donkey anti-mouse or anti-rabbit (LI-COR Biosciences).

Quantitative real-time PCR

RNA was extracted using the Aurum Total RNA Mini Kit (Bio-Rad, Hercules, CA, USA) following manufacturer's protocol. RNA quantity and purity (A_{260}/A_{280}) was determined using NanoDrop (Thermo Fisher Scientific). iScript™ advanced cDNA Synthesis Kit (Bio-Rad) was used with no modifications to obtain cDNA. 10ng of cDNA were used for each PCR reaction and SsoAdvanced™ Universal SYBR® Green Supermix (Bio-Rad) was used for the reaction mix with the primers; two technical replicates were used for each sample. Data was analysed using Bio-Rad CFX Maestro 2.3 Gene Study software, and expression normalized against multiple internal housekeeping genes was automatically generated. Files were exported to GraphPad Prism to be graphically presented. Primers were purchased from Integrated DNA Technologies and sequence is provided: **CD15** Fwd TGTGCATCTCCTTGACTGCC, Rvs GAAGAACCCCCTCCTTCAC; **RUNX1** Fwd TGAGCTGAGAAATGCTACCGC, Rvs ACTTCGACCGACAAACCTGAG; **SPI1** Fwd CCTGAGGGGCTCTGCATTGG, Rvs GTCCCAGTAATGGTCGCTATGG; **CDC6** Fwd CGGGTTCCGGCGAGG, Rvs GTTGTCATCGCCCAGACG; **DCPS** Fwd ACAAGAAGGCTGAAGCGGAC, Rvs CAAGTACAAGTCATCGAGCTGC; **MCM3** Fwd GCGCAGGAAAAACGAGAAGAG, Rvs AATGGAGGCCACAAAATCCTTT; **FHIT** Fwd CGGTCACAGGACTTTTTGCC Rvs TGGGAATGAAAGGCAGAGGG; **ISG15** Fwd CCTTCAGCTCTGACACC, Rvs CGAACTCATCTTTGCCAGTACA; **STAT1** Fwd CAGCTTGACTCAAAATTCCTGGA, Rvs TGAAGATTACGCTTGCTTTTCCT; **STAT2** Fwd CCAGCTTTACTCGCACAGC, Rvs AGCCTTGGAATCATCACTCCC; **IRF7** Fwd GCTGGACGTGACCATCATGTA, Rvs GGGCCGTATAGGAACGTGC; **IRF9** Fwd GCCCTACAAGGTGTATCAGTTG, Rvs TGCTGTCGCTTTGATGGTACT; **RPL13A** Fwd CCTGGAGGAGAAGAGGAAAGAGA Rvs TTGAGGACCTCTGTGTATTTGTCAA; **RPL37A** Fwd GATCTGGCACTGTGGTTCCT, Rvs CAGCGGAAGTGGTATTGTACG; **YWHAZ** Fwv ACTTTTGGTACATTGTGGCTTCAA, Rvs CCGCCAGGACAAACCAGTAT; **HPRT1** Fwd TGACACTGGCAAAACAATGCA, Rvs

GGTCCTTTTCACCAGCAAGCT; **TBP** Fwd CACGAACCACGGCACTGATT, Rvs TTTTCTTGCTGCCAGTCTGGAC; **ACTB** Fwd CTGGAACGGTGAAGGTGACA, Rvs AAGGGACTTCCTGTAACAATGCA.

Gene silencing and Over-expression

Lentiviral vectors were purchased from VectorBuilder for FHIT knock-down (in OCI-M1), FHIT over-expression (in OCI-AML2, OCI-AML3, MV4-11, MOLM-13) and STAT5B knock-down (in MOLM-13 and OCI-AML3). Vector control (EV) (VectorBuilder, Chicago, IL, USA) were used as negative controls. An MOI of 5 was used for FHIT KD, while an MOI of 1 was used for the over-expression. For STAT5B KD a MOI of 10 was used. Cells were transfected using Polybrene (VectorBuilder) and spinoculating at 300 rcf for 30 minutes using low acceleration speed. Selection with Puromycin (Sigma-Aldrich) or Blasticidin (Invivogen, Thermo Fischer Scientific) was done after 48 hours from transfection, and selected cells were expanded or frozen for downstream applications. Western Blot was used to test for efficiency of the transfection. Vectors names and target sequences are reported:

	**Vector name**	**Target sequence**
FHIT over-expression	pLV[Exp]-Bsd-CMV>3xFLAG/hFHIT[NM_002012.4]	
ORF Stuffer (EV)	pLV[Exp]-Bsd-CMV>ORF_Stuffer	
Scramble shRNA (EV)	pLV[shRNA]-Puro-U6>Scramble_shRNA#1	CCTAAGGTTAAGTCGCCCTCG
hFHIT shRNA #1	pLV[shRNA]-Puro-U6>hFHIT[shRNA#1]	TGTCCTTCGCTCTTGTGAATA
hFHIT shRNA #2	pLV[shRNA]-Puro-U6>hFHIT[shRNA#2]	TCATCTCACCATCCTGTATTC
hSTAT5B shRNA #1	pLV[shRNA]-Bsd-U6>hSTAT5B[shRNA#1]	CATCAGATGCAAGCGTTATAT

Cellular Thermal Shift Assay (CETSA®)

CETSA® Technology (Pelago Bioscience, Sweden) was used. Cells were plated in 24-well plates at 1x106 cells per well, then serial dilutions of RG3039 in complete medium were added to the cells. Cells

were harvested and resuspended in PBS (Thermo Fisher Scientific) with protease inhibitor, then incubate at 55°C or 64.3°C (tested previously) using a 3-minute melt-curve; leave at RT for 3 minutes, then incubate at -80°C for 10 minutes, thaw and repeat the heat/thaw cycle once more. Spin samples at 10000 rcf for 15 minutes at 4°C and proceed with Western blot protocol using a 4-12% Bis-tris Gel. After the procedure, membranes are developed on the LI-COR Odyssey CLx instrument and images were analysed in Image Studio Lite™ Software (LI-COR, v.5.5).

Flow Cytometry analysis of differentiation

Cells were seeded in 12-well plates at $5x10^5$ cells per well, then treated in duplicates with RG3039 at concentrations of 0.3, 1 and 3µM and incubated for 5 days. All-trans retinoic acid (ATRA) (Thermo Scientific) and 2,25-dihydrovitamin D3 (MedChemExpress) were used as positive controls, while DMSO was used as negative control. After treatment, cells were harvested and 50,000 cells per well were seeded in a 96-well plate in 100µL; two technical replicates were used for each sample. After washing twice with PBS, 100µL of LIVE/DEAD® Fixable Near-IR Dead cell stain (Thermo Fisher Scientific) diluted 1:1,000 in PBS were added to the wells and incubated for 10 minutes at 4°C in the dark. After washing with PBS supplemented with 2% FBS, 100µL of Fc Receptor Binding Inhibitor (eBioscience™, Thermo Fischer Scientific) diluted 1:50 was added to the wells, and incubated for 10 minutes as previously. 10µL of antibody suspension in PBS with 2% FBS was added to the wells and incubated an additional 20 minutes at 4°C in the dark. Plates were washed 3 times with PBS with 2% FBS, then 50µL of Fixation Buffer (Biolegend, San Diego, CA, USA) were added to each well, incubated for 15 minutes at room temperature, then washed away twice. Cells were resuspended in PBS with 2% FBS for the flow cytometry analysis. Data were obtained with CytoFLEX flow cytometer (Beckman Coulter, Indianapolis, IN, USA) an analysed with FlowJo™ Software v. 10.9 (Becton Dickinson, Franklin Lakes, NJ, USA). For compensation, ArC™ amine reactive compensation bead kit (Thermo Fisher Scientific) and AbC™ Total Antibody Compensation bead kit (Thermo Fisher Scientific) was used, following the compensation protocol of the instrument.
Antibodies used: CD15 Monoclonal Antibody (MMA), APC; IgM Mouse, APC, Clone: 11E10, Isotype Control (eBioscience); Brilliant Violet 421™ anti-human CD86 Antibody; Brilliant Violet 421™ Mouse IgG1, κ Isotype Ctrl; Alexa Fluor 488 anti-human CD14 antibody; Alexa Fluor® 488 Mouse IgG1, κ Isotype Ctrl; Anti-CD11b Mouse Monoclonal Antibody, FITC; Anti-IgG1, k Isotype Ctrl Mouse Monoclonal Antibody, FITC (Biolegend).

Flow Cytometry analysis of apoptosis

Dead cell apoptosis kit with Annexin V Alexa Fluor 488 and Propidium Iodide (PI) (Thermo Fisher Scientific) was used to assess the effect of RG3039 on OCI-AML2 and OCI-AML3. The manufacturer's protocol was followed, and samples were analysed on the Cytoflex (Beckman Coulter), then results were analysed using FlowJo™ software. Compensation was performed at the same time using cells stained with single fluorophores and non-stained cells.

Tumor Zebrafish Xenograft (ZTX®) in-vivo model

The zebrafish adults used in this study to generate embryos were raised and maintained in the zebrafish facility at Linköping University, Linköping, Sweden. All experiments were approved by Linköping animal research ethical committee. Zebrafish embryos were collected and incubated in E3 embryo medium (containing 0.286 g NaCl, 0.048 g CaCl2, 0.081 g MgSO4 and 0.0126 g KCl per liter, pH 7.2) supplemented with 0.2 mM PTU (E3/PTU) at 28.5 °C until 48 hours post fertilization.

AML PDX models were profiled elsewhere and procured as cryopreserved tumor tissue samples from Charles River Laboratories (1). Tumor tissues were thawed and washed gently by inversion with 10 mL of RPMI-1640 medium supplemented with 10% FBS (RPMI/10% FBS), then, the tissue samples were minced using surgical scissors to obtain ~ 1–3 mm^3 pieces. Five millilitres of PDX-disruptor Mix (Cat # EM_A1-007, BioReperia, Sweden) were added to the minced tissue and transferred to a Gentle MACS™ C-tube and mechanically dissociated using a gentleMACS™ Octo Dissociator for 30 min at 37 °C. The resulting single-cell suspension was washed with RPMI/10% FBS, filtered and finally labelled with 10 µg/mL DiI dye (VWR cat # APOSBIB6289) in RPMI/2% FBS for 30 min at 37 °C. After labelling, washing, and filtering, the sample was evaluated for cell viability using trypan blue viability exclusion dye.

DiI-labelled cells were implanted in the yolk sac of 2 days old zebrafish embryos. Injected embryos were selected under a fluorescent stereoscope model M205 FA (Leica Microsystems CMS GmbH), and primary tumors were photographed using the Leica Application Suite X (LAS X) software v3.7.1.21655 (Leica Microsystems CMS GmbH). Images were acquired using a K5 camera (Leica) with resolution (h x v) 2048 × 2048 pixels and pixel size (h x v) 6.5 × 6.5 µm, at an image magnification of 100x with no binning. Embryos were randomly sorted into experimental groups of 20 embryos per group and incubated for 72 h at 35.5 °C with E3/PTU water containing RG3039 at 3, 0.3, or 0.03 µM or vehicle.

Images were obtained right after implantation (day 0) and after 72 h incubation (day 3) and analysed by using the HuginMunin software v2.10.1.0 (BioReperia AB, Linköping, Sweden). Tumor sizes were analysed as the area of labelled tumor cells at day 0 and day 3. Relative tumor size was calculated by dividing the tumor area at day 3 by the area at day 0 in the same embryo and multiplying by 100. Data was normalized to the control group.

EdU incorporation assay

Click-iT™ Plus EdU Flow Cytometry Assay Kit (Thermo Fischer Scientific) protocol was used. Briefly, 100µL Click-iT fixative was added to 100µL cell suspension and incubated for 15 minutes. Cells were washed twice and resuspended in 1X Click-iT Saponin-based permeabilization wash reagent, the incubated for 15 minutes. 500µL of Click reaction mix were added for 30 minutes. After washing with 1X Saponin-based wash, 500µL of Propidium Iodide (PI) solution was added and incubated for 30 minutes. 633 and 488nm excitation was used to detect Alexa Fluor 647 azide and PI, respectively using CytoFLEX (Beckman Coulter).

Cellular Fractionation

The protocol from Yu and colleagues (2) was used to perform isolation of cytosolic and nuclear fraction. Briefly, cell pellet was lysed with subcellular fractionation (SF) buffer on tube roller for 30 minutes at 4°C, then centrifuged at 4°C. Supernatant was centrifuged for 10 minutes at 12’000 g at 4°C to remove cell debris, the collected in a new tube as cytosolic fraction. Pellet was washed once with SF buffer then resuspended in nuclear lysis (NL) buffer and agitated for 15 minutes at 4°C, then centrifuged for 5 minutes at 12’000 at 4°C to obtain the nuclear fraction.

Luciferase assay

For Interferon activity assays, THP1-Dual™ cells and STING KO cells (InvivoGen, Toulouse, France) were cultured in a basal media of RPMI-1640 (Thermo Fischer Scientific) supplemented with 10% heat-inactivated fetal bovine serum (FBS). The cells were maintained in basal media containing 100 µg/mL zeocin + 10 µg/mL blasticidin, prior to being exchanged into basal media for experiments. The THP1-Dual™ WT and STING KO cells were plated into a 96-well plate at 0.7-0.8 × 10^5 cells/well and stimulated with IFN-β (1ng/mL; Thermo Fischer Scientific) and cGAMP (20µg/mL; InvivoGen) controls for 24 or 48 h. Experimental wells were simultaneously treated with RG3039, using 3-fold dilutions at a

starting concentration of 20µM. At end of experiment, 20 µL of supernatant was transferred from culture wells to a new 96-well white plate and 50 µL of QUANTI-Luc™ 4 Lucia/Gaussia, a Lucia and Gaussia luciferase detection reagent was added with luminometer with the following parameters: 50 µL of injection, end-point measurement with a 4 second start time and 0.1 second reading time. The luminescence value was exported on Excel and normalized to DMSO control.

Cytospin and histochemical stain

Thermo Scientific Cytospin 4 Centrifuge was used at 400rpm for 5 minutes to fix cells on glass slides. Then, May-Grünwald Giemsa staining procedure from Sigma Aldrich was followed. Slides were imaged using Pannoramic Scan II Instrument (3DHISTECH, Budapest, Hungary), FIJI was used to analyse the images (3).

Clonogenic assay

Human Colony-Forming Unit (CFU) Assay was performed using MethoCult™ (STEMCELL Technologies, Vancouver, Canada) media and following STEMCELL Technologies protocol. Briefly, 1'000 cells were diluted 1:10 in MethoCult™ media, then dispensed in 35mm Petri dishes in duplicates. Cells were incubated in a humid chamber at 37°C for 14 days, without perturbations. Colony number and morphology were assessed by brightfield microscope. Optical readout was validated staining colonies with PBS containing 0.8% nitro blue tetrazolium chloride (NBT) for 16 hours and scanning using LI-COR Odissey CLx. Fluorescent signal in 800nm channel was quantified using Studio Lite™ Software (LI-COR, v.5.5).

Statistical analysis

GraphPad Prism v 10.3.1 was used for statistical analysis. All data are presented as mean ± standard deviation (SD) from ≥3 biological replicates, the specific sample size is detailed in the corresponding figure legends. The sample size for zebrafish experiments was determined based on preliminary research (n=20); zebrafish embryos that did not survive due to treatment-unrelated causes were not considered for tumor size quantification. To assess the significance between groups unpaired two-sided t-test was used, normality and homoscedasticity were controlled. P values are indicated by asterisks, *p< 0.05; **p< 0,01; ***p< 0,005; ****p< 0.001. To assess correlation relevance Pearson R square was used, and values are indicated in the figure graphs. Log-rank (Mantel-Cox) test of Kaplan-Meier survival

curves was used. The investigators were not blinded to the allocation through the experimental procedures and outcome assessment.

Data Availability Statement

All data generated during this study are included in this published article. Datasets analysed in *Figure 1A* are available from DepMap, Broad (2024). DepMap 24Q4 Public. Figshare+. Dataset. https://doi.org/10.25452/figshare.plus.27993248.v1. Datasets analysed in *Figure 1F* are extracted from https://ww.cbioportal.org (4).

References

1. Schueler J, Greve G, Lenhard D, Pantic M, Edinger A, Oswald E, et al. Impact of the injection site on growth characteristics, phenotype and sensitivity towards cytarabine of twenty acute leukaemia patient-derived xenograft models. Cancers (Basel). 2020 May 25;12(5):1349.

2. Yu Z, Huang Z, Lung ML. Subcellular Fractionation of Cultured Human Cell Lines. Bio Protoc. 2013 May 5;3(9).

3. Schindelin J, Arganda-Carreras I, Frise E, Kaynig V, Longair M, Pietzsch T, et al. Fiji: An open-source platform for biological-image analysis. Nat Methods. 2012 Jul;9(7):676–82.

4. Bottomly D, Long N, Schultz AR, Kurtz SE, Tognon CE, Johnson K, et al. Integrative analysis of drug response and clinical outcome in acute myeloid leukemia. Cancer Cell. 2022 Aug 8;40(8):850-864.

Supplementary Figure 1. **A** Quantification of FHIT and DCPS relative protein expression in AML cell panel. Data is shown as mean ± SD of log-transformed values normalized to β-actin (*n*=2). **B** Gene expression levels of *FHIT* and *DCPS* measured by RT-qPCR and normalized to *HPRT1*, *RPL37A* and *YWHAZ*, shown as mean ± SD (*n*=2). **C** Nonlinear regression curves of viability of AML cell panel upon treatment with RG3039. DMSO and Benzethonium Chloride (BzCl) were used as negative and positive controls, respectively. Dotted lines represent the most sensitive cell lines. IC50 values were quantified in **D** and expressed as mean ±SD (*n*=3). **E** Cellular Thermal Shift Assay (CETSA®) evaluation of target stabilization. Denaturing temperature was previously determined for each protein. DCPS shows a full target stabilization with RG3039, while no engagement is evident for FHIT. MOLM-16 cell line was used. **F** Immunoblot analyses of FHIT shRNA knock-down in OCI-M1 using two constructs (shRNA #1, shRNA #2). Non-transduced parental cells (par) and non-targeting vector (EV) were used as controls. Calculated IC50 are shown as mean ±SD (*n*=4); $^*p < 0.05$, unpaired t test. **G** Immunoblot analyses and quantification of lentiviral over-expression of 3Xflag-FHIT in AML cell lines (FHIT-OE in blue); empty vector (EV in red) and parental cell line (par) were used as controls. **H** Immunoblot analyses of STAT5B protein levels in MOLM-13 EV and FHIT-OE treated with RG3039 for 24 hours; GAPDH was used for signal normalization. Quantification is shown as mean ±SD of relative expression to DMSO (*n*=3); $^{***}p < 0.005$, $^{****}p < 0.001$, unpaired t test. **I** Scatter plot of *DCPS* and *FHIT* gene expression levels (TPM) for the patient-derived AML samples from the Charles River Laboratories biobank used for xenografting in the Zebrafish embryos using Bioreperia's ZTX® platform. Two samples were selected with low FHIT expression and positive for DNMT3A and FLT3 mutations; two controls were selected with high FHIT expression and DNMT3A and FLT3 wild-type genotype.

Supplementary Figure 2. **A** Bar chart showing mean ± SD of OCI-AML3 gene expression for *CDC6* and *MCM3* after 6-hour treatment with RG3039 relative to DMSO negative control (*n*=4). Expression was normalized to *RPL37A* and *HPRT1* housekeeping genes; $^*p < 0.05$, unpaired t test. **B** Scatterplot showing Pearson correlations between *DCPS* and *MCM3* (left, R=0.503) or *CDC6* (right, R=0.285) mRNA levels in patients from cBioPortal OHSU AML repository, expressed as log2 RNA Seq RPKM; $p < 0.0001$. **C** Immunoblot analyses of CBP80 expression in nuclear and cytosolic fractions from OCI-AML3 treated with RG3039 for 24 hours. Quantification is shown in **D** as mean ±SD of relative expression to DMSO; α-tubulin (cytosolic) or HH3 (nuclear) were used for signal normalization (*n*=4);

*$p < 0.05$, **$p < 0.01$, ***$p < 0.01$, unpaired t test. **E** Bar chart of early apoptosis in EV and FHIT-OE OCI-AML3 cells induced by RG3039. Percentage of Annexin V positive cells was calculated by flow cytometry and expressed as mean ±SD (n=3). **F** Representative pictures of OCI-AML3 EV and FHIT-OE colonies with granulocyte-macrophage (GM) morphology upon treatment with RG3039 identified in MethoCult™ media after 14 days incubation. Scale bar is 100μm.

Supplementary Figure 1

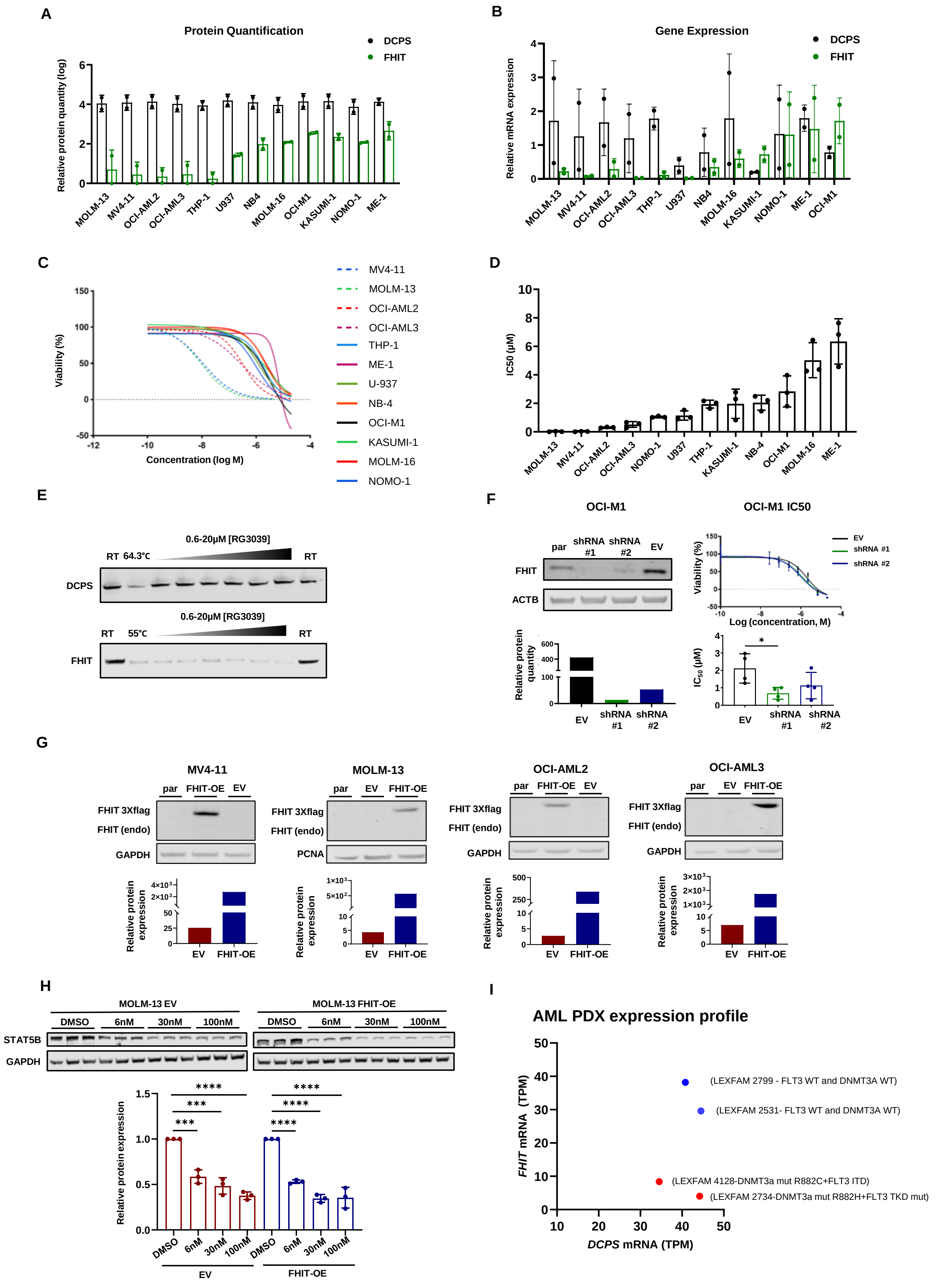

Supplementary Figure 2

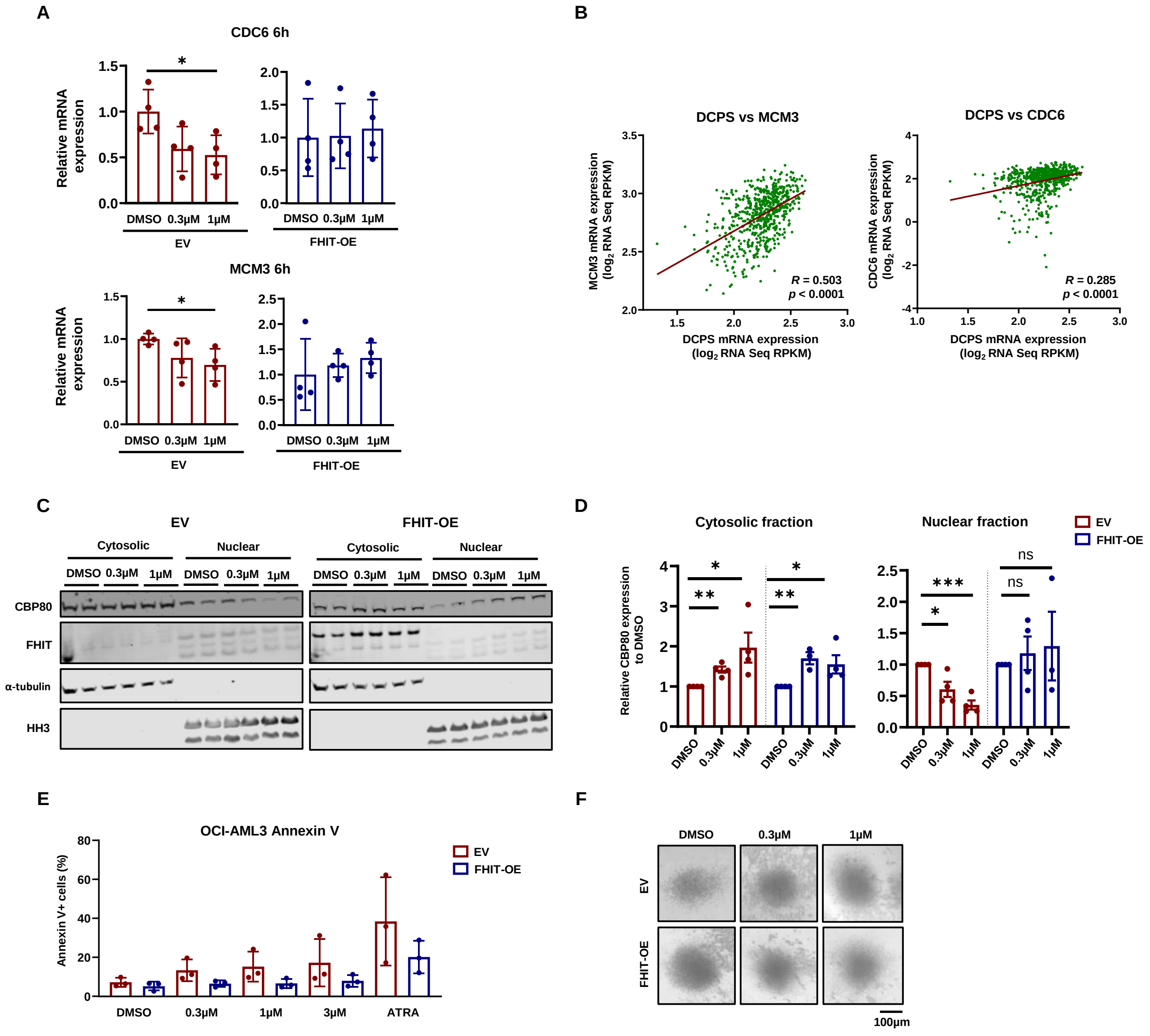

Supplement: Supplementary file 1 — Supplementary Information [file 41375_2025_2661_MOESM1_ESM.pdf]
